# Cardioembolic Stroke in a Patient with Coronavirus Disease of 2019 (COVID-19) Myocarditis: A Case Report

**DOI:** 10.5811/cpcem.2020.6.47856

**Published:** 2020-06-22

**Authors:** James S. Ford, James F. Holmes, Russell F. Jones

**Affiliations:** UC Davis Health, Department of Emergency Medicine, Sacramento, California

**Keywords:** COVID-19, SARS-CoV-2, myocarditis, thromboembolic stroke

## Abstract

**Introduction:**

There is a growing body of literature detailing coronavirus 2019 (COVID-19) cardiovascular complications and hypercoagulability, although little has been published on venous or arterial thrombosis risk.

**Case Report:**

In this report, we present a single case of cardioembolic stroke in the setting of COVID-19 related myocarditis, diagnosed via cardiac magnetic resonance imaging and echocardiography. COVID-19 infection was confirmed via a ribonucleic acid polymerase chain reaction assay.

**Conclusion:**

Further research is needed to evaluate the hypercoagulable state of patients with COVID-19 to determine whether prophylactic anticoagulation may be warranted to prevent intracardiac thrombi and cardioembolic disease in patients with COVID-19 related myocarditis.

## INTRODUCTION

The first cases of coronavirus of 2019 (COVID-19) were reported in Wuhan, China, in December 2019.^1^ While the full spectrum of clinical disease that the virus can cause has yet to be elucidated, a growing body of literature is emerging detailing various cardiovascular complications, ranging from myocardial injury with mild troponin elevations to fulminant myocarditis.^2^,^3^ Elevations in cardiac biomarkers such as B-type natriuretic peptide (BNP, 27.5% of patients) and cardiac troponin (7–17%) are common, with the latter being associated with worsening disease severity, intensive care unit status, and mortality.^4^–^6^ Additionally, cardiac dysrhythmias from a variety of etiologies have been reported.^2^ In a case series of 150 patients with confirmed COVID-19, 7% of all deaths were attributed to myocarditis with ensuing circulatory collapse.^7^

Derangements of coagulation laboratory studies have also been reported from COVID-19, including elevations of D-dimer, modulation of fibrinogen (high in early disease, low in advanced disease), and modulation of prothrombin time and partial thromboplastin time, with over 70% of non-survivors in one study meeting criteria for disseminated intravascular coagulation.^8^ While rates of venous thromboembolism have not been reported for COVID-19, severe acute respiratory syndrome, coronavirus 1, (SARS-CoV-1)(2013) was associated with ischemic stroke, deep venous thrombosis and pulmonary embolism, making it probable that similar complications are possible with the 2019 novel virus.^9^ A recent case series identified seven cases of acro-ischemia in patients without evidence of shock and not on vasopressor support, providing early evidence of hypercoagulability in COVID-19 infection.^10^ In this report, we present a single case of cardioembolic stroke in the setting of COVID-19 related myocarditis.

## CASE REPORT

A 53-year-old male with a past medical history significant only for hyperlipidemia, was brought in by ambulance to the emergency department (ED) with a six-day history of malaise and fever (T_max_ 101°Fahrenheit [F]), and one day of cough. The day before presentation, he was seen in an outside ED and discharged home with a diagnosis of viral upper respiratory syndrome. On presentation to our ED the next day, he reported a brief episode of chest pain with palpitations that resolved spontaneously after 30 minutes. He denied shortness of breath, nocturnal dyspnea or lower extremity swelling. Vitals at triage were notable for temperature of 100.2°F, heart rate 140 beats per minute, blood pressure 97/55 millimeters of mercury, respiratory rate 16 breaths per minute, and oxygen saturation of 100%.

Exam was notable for diaphoresis, with clear breath sounds bilaterally, tachycardia with irregular pulse with no murmurs, and no lower extremity edema. Electrocardiogram (ECG) demonstrated a wide-complex, irregular tachycardia with a left bundle branch block (LBBB) morphology not meeting modified Sgarbosa criteria (Concordant ST elevation > 1millimeter [mm] in leads with a positive QRS complex; concordant ST depression > 1 mm in V1–V3; discordant ST elevation [or depression] relative to the preceding S-wave [or R-wave] with 1) at least 1 mm of ST elevation (or depression) AND 2) an ST/S(R) ratio ≤ -0.25) that was favored to be atrial fibrillation with rapid ventricular response or sinus tachycardia with frequent premature atrial contractions, as well as a corrected QT interval (QTc) of 563 (Reference [Ref]: ≤ 440, in males).^11^ No comparison ECG was available. Laboratory work-up was significant for hypokalemia (K+ 2.8 milliequivalents per liter [mEq/L] [Ref: 3.3–5.0 mEq/L]), normal creatinine, white blood cell count of 5.5 thousand per cubic millimeter [K/mm^3^] [Ref: 4.5–11 K/mm^3^] with lymphopenia (absolute lymphocyte count, 0.7 K/mm^3^ [Ref: 1.0–4.8 K/mm^3^]), mild transaminitis (aspartate aminotransferase 63 units/L [Ref: 15–43 units/L], alanine transaminase 72/L [Ref: 6–63 units/L), negative serial high-sensitivity troponin T (< 99 percentile) and BNP 588 picogram (pg)/mL (Ref: 1–100pg/mL). Chest radiograph (CXR) showed a left lower lobe consolidation. His potassium was replaced, he was given an amiodarone load, started on ceftriaxone/azithromycin for presumed community-acquired pneumonia, and admitted to the cardiology service.

During his hospitalization, he converted to normal sinus rhythm with electrolyte replacement and amiodarone load, but LBBB morphology and prolonged QTc persisted. Azithromycin was changed to doxycycline due to concerns of potentially worsening QTc prolongation from azithromycin. Rapid flu test, respiratory viral panel (RVP), legionella urine antigen, and blood cultures were all negative. Transthoracic echocardiography (TTE) was performed which showed mild left ventricular (LV) dilation with hypokinesis (ejection fraction 15%). There was no comparison TTE available. Cardiac catheterization did not reveal significant coronary artery disease. Cardiac magnetic resonance imaging (MRI) with contrast confirmed LV dilation with global hypokinesis, increased T2 signal, hyperemia, and edema consistent with viral myocarditis. In the setting of these MRI findings, the patient’s CXR infiltrate, and the absence of an additional viral etiology for myocarditis (negative RVP), radiology recommended dedicated COVID-19 testing.

**CPC-EM Capsule**

What do we already know about this clinical entity?

Coronavirus disease 2019 (COVID-19) can cause cardiovascular complications, including myocarditis. New evidence is emerging describing the hypercoagulability of COVID-19 patients.

What makes this presentation of disease reportable?

COVID-19 myocarditis and left ventricular thrombus formation with cardioembolic stroke has not been previously reported.

What is the major learning point?

COVID-19 myocarditis with associated left-heart dilation and hypercoagulability may predispose patients to cardioembolic stroke, especially in patients with underlying cardiomyopathy.

How might this improve emergency medicine practice?

Understanding clinical sequelae related to COVID-19 will help tailor diagnostics and therapeutics related to cardiovascular complications of infection.

The patient was tested using a qualitative ribonucleic acid polymerase chain reaction assay (via nasopharyngeal swab) and resulted positive. However, since LV dilation (defined as LV end diastolic diameter [LVEDD] >3.3 centimeters per meter squared [cm/m^2^]) is only present in approximately 50% of patients with acute myocarditis, and the range of LVEDD reported in one study of myocarditis patients with LV dilation was 3.4–6.1cm/m^2^, the patient’s initial LVEDD (5.96 cm/m^2^) approached the upper range for LV dilation expected in myocarditis, leading the cardiology service to suspect a chronic undiagnosed cardiomyopathy.^12^ The patient had no evidence of ischemic heart disease, history of alcohol or methamphetamine use, or any other obvious etiology of cardiomyopathy. Further investigation revealed that the patient had spent time in Mexico, so he was tested for Chagas disease. This was a send-out lab and would not result for several days. The patient was discharged in stable condition on hospital day four with new prescriptions for metoprolol succinate, losartan and spironolactone.

Three days later, the patient returned to the ED with acute expressive aphasia without other neurological deficits. MRI and MR angiography of the brain revealed an acute left middle cerebral artery stroke involving Broca’s area. He was treated with tissue plasminogen activator and admitted to the neurology service. ECG demonstrated a stable LBBB with no acute ST-T wave changes. Troponin T was elevated at 66 nanograms per liter (ng/L) (Ref: <19ng/L), and peaked at 373ng/L, 11 hours later. Emergent computed tomography angiography of the neck, did not reveal any carotid lesions, but did show ground-glass infiltrates in bilateral lung apices, consistent with COVID-19 infection.^4^ CXR showed bilateral peripheral airspace opacities (left greater than right). Repeat TTE showed a new LV thrombus (not visualized on TTE or cardiac MRI from previous hospitalization) and worsening LV dilation (diastolic diameter increased from 5.96 cm to 6.53 cm, compared to previous echocardiography six days earlier). He was started on anticoagulation therapy with warfarin with a heparin bridge, and transferred to an outside hospital for continued care and rehabilitation.

The patient’s *Trypanasoma cruzi* (T. *cruzi)* immunoglobulin G (IgG) was found to be positive at 1.8, suggesting active or past infection. The patient was notified of this finding, and infectious disease follow-up was arranged.

## DISCUSSION

While the patient’s presentation was consistent with acute viral myocarditis, the presence of *T. cruzi* IgG antibodies confounded the clinical picture. Since it is possible that the patient had undiagnosed Chagas cardiomyopathy, it is difficult to know whether the patient’s presentation was truly related to COVID-19 myocarditis or simply was related to chronic pre-existing heart failure. However, myocardial fibrosis, a marker of Chagas cardiomyopathy that is detected as delayed gadolinium enhancement on cardiac MRI, was not seen in our patient, making Chagas cardiomyopathy less likely.^13^ Furthermore, acute worsening of LV dilation and rising troponin levels, suggested an acute, rather than chronic process, making viral (COVID-19) myocarditis more likely.

In the setting of a newly diagnosed LV thrombus, the most likely source of the patient’s stroke was cardioembolic. Transesophageal echocardiography (TEE) is the gold standard for diagnosing intracardiac thrombi. However, cardiac MRI has been found to be both more sensitive and specific than TEE for detecting LV thrombus, making it unlikely that the LV thrombus was present during the initial hospitalization, and instead, more likely that the thrombus formed during the three days between discharge and re-hospitalization.^14^ While it is possible that the patient had undiagnosed paroxysmal atrial fibrillation that predisposed him toward forming a LV thrombus, this seems unlikely given that his irregular rhythm converted to sinus after electrolyte correction, and he did not re-enter an irregular rhythm during the same hospitalization, or during subsequent re-hospitalization. Furthermore, if he indeed did have atrial fibrillation, his CHA_2_DS_2_-VASc score would have been one (one point for newly diagnosed heart failure, annual stroke risk 0.6%), making it unlikely that a ventricular thrombus could have formed and embolized in three days.^15^

The prevalence of LV thrombus in patients with dilated cardiomyopathy with reduced ejection fraction and sinus rhythm, is as high as 13%, with increasing LV size being independently associated with LV thrombus.^16^,^17^ Since complete coagulation parameters were not initially obtained, it is difficult to quantify his level of hypercoagulability. Nonetheless, it is likely that this hypercoaguable state, in conjunction with acute myocarditis and worsening LV dilation, predisposed the patient to LV thrombus formation and cardioembolic stroke.

## CONCLUSION

Myocarditis is a serious complication of COVID-19 infection and may predispose patients to further cardiovascular injury, such as cardioembolic stroke. Further research is needed to evaluate the full scope of cardiovascular complications in order to better inform treatment. Prophylactic anticoagulation should be considered in high-risk patients at risk for venous and arterial thromboembolism.

## References

[1] World Health Organization (2020). Pneumonia of unknown cause — China.

[2] Driggin E, Madhavan MV, Bikdeli B (2020). Cardiovascular considerations for patients, health care workers, and health systems during the coronavirus disease 2019 (COVID-19) Pandemic. J Am Coll Cardiol.

[3] Madjid M, Safavi-Naeini P, Solomon SD (2020). [Ahead of Print]. Potential effects of coronaviruses on the cardiovascular system: a review. JAMA Cardiol.

[4] Huang C, Wang Y, Li X (2020). Clinical features of patients infected with 2019 novel coronavirus in Wuhan, China. Lancet.

[5] Zhou F, Yu T, Du R (2020). Clinical course and risk factors for mortality of adult inpatients with COVID-19 in Wuhan, China: a retrospective cohort study. Lancet.

[6] Wang D, Hu B, Hu C (2020). Clinical characteristics of 138 hospitalized patients with 2019 novel coronavirus-infected pneumonia in Wuhan, China. JAMA.

[7] Ruan Q, Yang K, Wang W (2020). Clinical predictors of mortality due to COVID-19 based on an analysis of data of 150 patients from Wuhan, China. Intensive Care Med.

[8] Tang N, Li D, Wang X (2020). Abnormal coagulation parameters are associated with poor prognosis in patients with novel coronavirus pneumonia. J Thromb Haemost.

[9] Umapathi T, Kor AC, Venketasubramanian N (2004). Large artery ischaemic stroke in severe acute respiratory syndrome (SARS). J Neurol.

[10] Zhang Y, Cao W, Xiao M (2020). Zhonghua Xue Ye Xue Za Zhi.

[11] Smith SW, Dodd KW, Henry TD (2012). Diagnosis of ST-elevation myocardial infarction in the presence of left bundle branch block with the ST-elevation to S-wave ratio in a modified Sgarbossa rule. Ann Emerg Med.

[12] Pinamonti B, Alberti E, Cigalotto A (1988). Echocardiographic findings in myocarditis. Am J Cardiol.

[13] Rochitte CE, Oliveira PF, Andrade JM (2005). Myocardial delayed enhancement by magnetic resonance imaging in patients with Chagas’ disease: a marker of disease severity. J Am Coll Cardiol.

[14] Srichai MB, Junor C, Rodriguez LL (2006). Clinical, imaging, and pathological characteristics of left ventricular thrombus: a comparison of contrast-enhanced magnetic resonance imaging, transthoracic echocardiography, and transesophageal echocardiography with surgical or pathological validation. Am Heart J.

[15] Lip GYH, Nieuwlaat R, Pisters R (2010). Refining clinical risk stratification for predicting stroke and thromboembolism in atrial fibrillation using a novel risk factor-based approach: the Euro Heart Survey on Atrial Fibrillation. Chest.

[16] Bakalli A, Georgievska-Ismail L, Koçinaj D (2012). Left ventricular and left atrial thrombi in sinus rhythm patients with dilated ischemic cardiomyopathy. Med Arch.

[17] Bakalli A, Georgievska-Ismail L, Koçinaj D (2013). Prevalence of left chamber cardiac thrombi in patients with dilated left ventricle at sinus rhythm: the role of transesophageal echocardiography. J Clin Ultrasound.

